# Cross Talk Between Ferroptosis and Cerebral Ischemia

**DOI:** 10.3389/fnins.2020.00776

**Published:** 2020-08-06

**Authors:** Xu She, Bin Lan, Haomei Tian, Biao Tang

**Affiliations:** Department of Physiology, Hunan University of Chinese Medicine, Changsha, China

**Keywords:** ferroptosis, cerebral ischemia, iron metabolism, amino acid metabolism, lipid metabolism

## Abstract

Recently, ferroptosis has been revealed as a new form of regulated cell death. Distinct from apoptosis and necrosis, ferroptosis is evoked by iron-dependent lipid peroxidation. Furthermore, the metabolism of iron, lipids, and amino acids plays a significant regulatory role in ferroptosis, which can be reversed by glutathione peroxidase 4 and ferroptosis suppressor protein 1. Ferroptosis is implicated in the onset and development of numerous neurological diseases. Emerging studies have reported that ferroptosis induces and aggravates brain tissue damage following cerebral ischemia, whereas inhibition of ferroptosis dramatically attenuates induced damage. In this review, we have summarized the mechanistic relationship between ferroptosis and cerebral ischemia, including through iron overload, downregulation of glutathione peroxidase 4, and upregulation of lipid peroxidation. Although considerable attention has been paid to the effect of ferroptosis on cerebral ischemic injury, specific mechanisms need to be experimentally confirmed, including how cerebral ischemia induces ferroptosis and how ferroptosis deteriorates cerebral ischemia.

## Introduction

Ferroptosis is a type of regulated cell death dependent on iron. In 2012, ferroptosis was first used to describe cell death induced by erastin, a small molecule that inhibits cystine uptake and leads to reduced glutathione (GSH) production, as well as the inactivation of glutathione peroxidase 4 (GPX4) (Dixon et al., 2012). Ferroptosis relies on intracellular iron rather than other metallic elements and could be inhibited by iron chelators and antioxidants, such as vitamin E. Moreover, it is distinct from other forms of cell death, including apoptosis, necroptosis, and autophagy-dependent cell death, in terms of morphology, biochemistry, and genetics (Dixon et al., 2012). Furthermore, inhibitors of apoptosis, necroptosis, or autophagy are incapable of suppressing ferroptosis (Dolma et al., 2003).

The main characteristic of ferroptosis is death due to the accumulation of lethal levels of iron-dependent lipid peroxides (for review, see Michael and Brent, 2017). Previous studies have shown that ferroptosis has pathological roles in a wide variety of diseases, including neurodegenerative diseases, cancer, ischemia–reperfusion injury, stroke, and traumatic brain injury (for review, see Conrad et al., 2016; Newton et al., 2016; Li et al., 2017; Toyokuni et al., 2017; Zille et al., 2017).

## Distinct Morphological Features of Ferroptosis as Compared to Apoptosis, Necrosis, and Autophagy

Morphologically, ferroptotic cells exhibit typical features of necrosis, with no apoptosis features. Importantly, ferroptosis is accompanied by a series of morphological changes in the mitochondria, including cristae reduction, membrane coagulation, and rupture of the outer membrane, whereas in other forms of cell death, the mitochondria are usually swollen (Dixon et al., 2012). Moreover, in cancer cells treated with ferroptosis inducers, like erastin, the structural integrity of the nucleus is maintained, with no nucleation or chromatin edge observed. These differences in morphological features are important to distinguish ferroptosis from apoptosis, necrosis, and autophagy (Xie et al., 2016).

Biochemical processes related to ferroptosis include metabolisms involving iron, amino acids, or polyunsaturated fatty acids (PUFAs), as well as the biosynthesis of GSH, phospholipids, and nicotinamide adenine dinucleotide phosphate, and coenzyme Q_10_ (for review, see Stockwell et al., 2017).

## Iron, Amino Acid, and Lipid Metabolisms Are Deeply Involved in the Molecular Mechanisms of Ferroptosis

Notably, mechanisms associated with ferroptosis are complex and mainly include three factors: iron, amino acid, and lipid metabolisms. Iron is an essential element in ferroptosis. A robust increase in iron levels can trigger the accumulation of lipid peroxides and reactive oxygen species (ROS), resulting in ferroptosis (Yan and Zhang, 2019).

Amino acid metabolism is closely associated with ferroptosis. In most cases, the intake of cysteine occurs via cystine/glutamate reverse transporters (system xc-), which function in the intracellular environment to synthesize GSH and maintain the antioxidant activity of GPX4.

Lipoxygenase transforms esterified PUFAs located in the cell membrane into lipid peroxides (Yang et al., 2016). Reportedly, lipoxygenase is a crucial agent playing a direct role in ferroptosis (Yang et al., 2016). Coenzyme Q_10_, a downstream effector of β-hydroxy β-methylglutaryl-CoA (HMG-CoA) reductase, which synthesizes mevalonic acid (MVA), is an endogenous ferroptosis inhibitor possessing profound antioxidant effects in the intracellular environment (Shimada et al., 2016). Suppression of this coenzyme can potentiate the accumulation of lipid peroxides, resulting in cell death (Shimada et al., 2016). According to the latest research, *FSP1* represses lipid peroxidation by reducing coenzyme Q_10_ through NAD(P)H, thus eliciting an inverse role in the occurrence of ferroptosis (Bersuker et al., 2019; Doll et al., 2019). These results indicate that in ferroptosis, cell death is characterized by lipid peroxidation. Conceivably, the interventions of ferroptosis by targeting lipid peroxidation are reasonable.

### Iron Metabolism

Iron overload is a key event in ferroptosis, with Fe^2+^ generating lipid ROS through the Fenton reaction (for review, see Toyokuni et al., 2017). Furthermore, Fe^2+^ constitutes the lipoxygenase (LOX) catalytic subunit that initiates lipid peroxidation. Both these functions of Fe^2+^ are crucial for the onset of ferroptosis. There are two transport mechanisms for the entry of non-heme iron into cells: transferrin (TF)-dependent and TF-independent mechanisms. Several mechanisms occur independently of TF; for example, extracellular Fe^2+^ enters cells through membrane divalent metal transporter 1 (DMT1). However, most plasma Fe^3+^ exists in the complete form with TF and is transformed into holotransferrin (for review, see Hentze et al., 2010). After endocytosis, holotransferrin binds to TF receptor 1 (TFR1) on the cell membrane surface and subsequently moves to endosomes. In the acidic environment of the endosome, Fe^3+^ is released from TF and converted into Fe^2+^ through oxidation–reduction. Consequently, Fe^2+^ is transported to the labile iron pool in the cytoplasm through DMT1 on the endosome membrane, where it forms a complex that functions as a physiological or pathological element (for review, see Richardson and Ponka, 1997; Hentze et al., 2010). The secretion or storage of excessive Fe^2+^ in the ferritin complex blocks the formation of hydroxyl radicals from H_2_O_2_, thus preventing ROS production (for review, see Richardson and Ponka, 1997). Some Fe^2+^ ions are transported outside the cell membrane through ferroportin1 (FPN1) residing in the cell membrane; therefore, the intracellular iron concentration is considered appropriate under physiological conditions (Donovan et al., 2000).

### Amino Acid Metabolism

A study has shown that the entrance of cystine into the cytoplasm could be the mainstay of ferroptotic suppression, through the maintenance of intracellular GSH levels (Le et al., 2015). In contrast, the decrease in GSH synthesis contributes to ferroptosis (Philpott and Ryu, 2014). The translocation of extracellular cystine and intracellular glutamate by system xc- is driven by the high glutamate concentration in the intracellular environment, rather than ATP, at a ratio of 1:1, and the repression of system xc- directly results in ferroptosis (Dixon et al., 2012). Following the injury of brain tissue, system xc- is impacted by the high concentration of extracellular glutamate (Jose et al., 2014; for review, see Bridges et al., 2012). After crossing the cytomembrane, cystine is degraded into cysteine and, subsequently, cysteine is converted into γ-glutamyl-cysteine by glutamyl-cysteine ligase. Then, γ-glutamyl-cysteine combines with glycine to form GSH, which in turn is activated by glutathione synthase (for review, see Aoyama and Nakaki, 2015).

In recent years, the suppression of GPX4, a key anti-lipid peroxidation enzyme, has been considered a committed step of ferroptosis, either through a direct (such as via covalent inhibition of RSL3 and its related molecules; Yang et al., 2014) or indirect (such as via GSH depletion) manner, provoking the unacceptable upregulation of phospholipid–OH and alkyl oxygen free radicals, causing severe membrane damage and ferroptosis (Dixon et al., 2012; Yan and Zhang, 2019). GPX4 activates phospholipid–OH to phospholipid-H via a reduction reaction, with the consumption of two molecules of GSH (Yan and Zhang, 2019). Additionally, GSH is transformed into oxidized glutathione (Yan and Zhang, 2019). Oxidized glutathione is then reduced to GSH by the nicotinamide adenine dinucleotide phosphate-dependent glutathione reductase, entering the next cycle of reduction reaction (Leslie and Scott, 2018). The oxidized phospholipid–OH is converted into massive alkyl oxygen free radicals with highly reactivity initiated by intracellular free Fe^2+^ (for review, see Gaschler and Stockwell, 2016). The latter induces adverse effects on adjacent PUFAs through a cascade of chain reactions. Eventually, an extensive spectrum of membrane damage occurs, decreasing cell viability and even causing death (for view see Philpott and Ryu, 2014).

Furthermore, as a binding ligand for free Fe^2+^ in the labile iron pool, GSH can prevent Fe^2+^ from reacting with H_2_O_2_. Conversely, highly cytotoxic hydroxyl radicals are generated by the lack of GSH (for review see Philpott and Ryu, 2014).

### Lipid Metabolism

Lipid metabolism is closely related to ferroptosis (for review, see Stockwell et al., 2017). Considering that PUFAs are sensitive to oxidants, they function as primary substrates of lipid peroxidation related to ferroptosis, especially arachidonic acid (AA) and adrenic acid (AdA) (Yan and Zhang, 2019). First, PUFAs are esterified with membrane phospholipids, mainly phosphatidylethanolamine (PE). The esterification reaction is catalyzed by acyl-CoA synthetase long-chain family member 4 (ACSL4) and lysophosphatidylcholine acyltransferase 3 (LPCAT3), resulting in the formation of AA/AdA-PE. Next, LOX converts PE-AA/AdA into PE-AA/AdA-OH through oxidation, thereby inducing ferroptosis (Dixon et al., 2015; Yang et al., 2016; Doll et al., 2017; Kagan et al., 2017; Shintoku et al., 2017). Furthermore, the degree of ferroptosis can be determined by measuring the amount of peroxidized PUFAs and examining their subcellular localization (Stockwell et al., 2017). However, the specific mechanism through which lipid peroxidation mediates ferroptosis remains unknown (Eran et al., 2018).

## Ferroptosis Exerts Profound Regulatory Effects in Cerebral Ischemic Brain Injury

The relationship between ferroptosis, as a fundamental form of cell death in the nervous system, and neurological diseases has been reported in several diseases, including Alzheimer’s disease, Parkinson’s disease, Huntington’s disease, amyotrophic lateral sclerosis, Friedreich’s ataxia, traumatic brain injury, and periventricular leukomalacia (Chen et al., 2015; Do Van et al., 2016; Gascón et al., 2016; for review, see Skouta et al., 2014; Guiney et al., 2017; Hambright et al., 2017; Li et al., 2017; Zille et al., 2017).

Ischemic stroke (IS) is a major cause of death and disability worldwide. It is characterized by the sudden obstruction of local blood flow to the brain and results in irreversible damage and deficiency of nerve function (for review, see Lee et al., 1999). There are multiple mechanisms involved in ischemic neuronal injury, among which oxidative stress is important in the progression of ischemic injury. In ischemic brain tissue, the defense ability of the antioxidant system is weakened owing to ROS elevation, followed by the excessive production of oxygen free radicals, deactivation of antioxidant enzymes, and antioxidant depletion. This results in multiple deleterious events, including peroxidation of lipids and proteins, damage to nuclear DNA, and cell death (for review, see Chan, 2001). Moreover, it has been shown that oxidative damage in the ischemic brain tissue is continuous and even spreads to surrounding healthy tissues; however, treatment with antioxidants can noticeably attenuate cerebral ischemic damage (Imai et al., 2003). A recent study has reported that oxidative stress injury can induce ferroptosis and leads to neuronal death in neurodegenerative diseases (Chuanhong et al., 2018) and that BID plays an important role in the ferroptosis mechanism-induced oxidative stress (Neitemeier et al., 2017) and is hence possibly linked with cerebral ischemic damage. However, the role of ferroptosis remains unclear in cerebral ischemia and further research is needed. Multiple studies have revealed that ferroptosis plays a regulatory role in ischemic brain damage, mainly in neurons. However, the suppressive intervention of ferroptosis can exert neuroprotective effects (DeGregorio-Rocasolano et al., 2019). These findings imply that ferroptosis possesses the potential to serve as a therapeutic target for cerebral ischemia (Cheah et al., 2006; Gubern et al., 2013; Tuo et al., 2017; Yigitkanli et al., 2017).

Reportedly, ferroptosis exerts profound regulatory effects during ischemia–reperfusion brain injury. Additionally, ferroptosis has been detected in mouse models of cerebral ischemia. Administration of the ferroptosis inhibitors, liproxstatin-1 or ferrostatin-1 (Fer-1), after reperfusion, generated a markedly smaller infarct, thus alleviating cerebral injury (Tuo et al., 2017). Furthermore, studies have revealed that inhibition of ferroptosis can reduce ischemic brain damage in rats, suggesting that ferroptosis mediates the onset and development of IS and exacerbates cerebral injury (Alim et al., 2019; Guan et al., 2019; Lan et al., 2020).

Moreover, the inflammatory mechanism is probably responsible for the aggravation of cerebral ischemic injury mediated by ferroptosis. Typically, ferroptosis exerts a strong pro-inflammatory effect through the release of pro-inflammatory mediators, and this inflammatory response is the key mechanism of cerebral ischemic injury (for review, see Linkermann et al., 2014b). Although research in this field is limited, a recent review indicated that ferroptosis plays an important role in inflammation (Sun et al., 2020) and, reportedly, inflammation is activated in ferroptotic tissues in mouse kidney injury models (Linkermann et al., 2014a). Additionally, the relationship between inflammation and dead neurons was demonstrated in the GPX4-depleted model (Hambright et al., 2017). Both the abovementioned findings provided an experimental basis for the hypothesis.

## Potential Regulatory Mechanisms of Ferroptosis in Cerebral Ischemia

**FIGURE 1:**
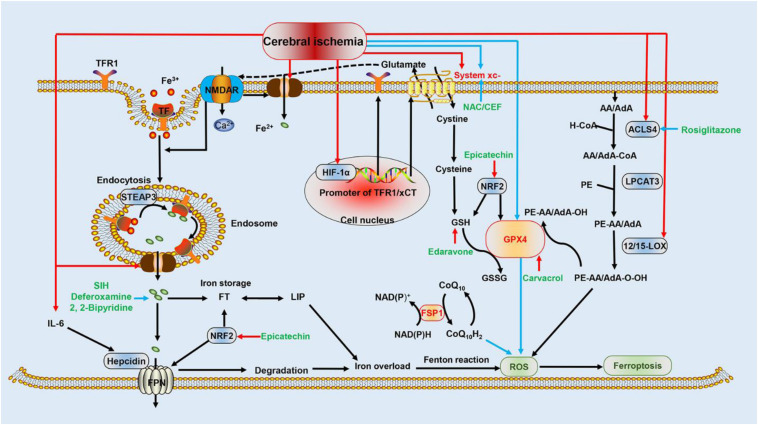
Schematic representation of ferroptotic mechanisms following cerebral ischemia. This figure summarizes the potential mechanism of cerebral ischemia in regulating ferroptosis and the molecular mechanisms of ferroptosis: (i) the red arrows indicate that HIF-1α, DMT1, ACSL4, 12/15-LOX, system xc-, and IL-6 are upregulated by cerebral ischemia, which promotes ferroptosis and worsens cerebral ischemia injury; (ii) the blue arrows indicate that system xc- and GPX4 are repressed during cerebral ischemia, promoting ferroptosis and potentiating damage; (iii) the additional FSP1 pathway inhibits the formation of ROS through CoQ_10_H_2_, and the Nrf2 pathway represses ferroptosis by attenuating iron overload and oxidative damage; (iv) black arrows represent the molecular mechanisms of ferroptosis; and (v) green font indicates drugs that inhibit ferroptosis and interfere with ischemic brain injury. Blue arrows indicate inhibition, and red arrows indicate stimulation. HIF-1α, hypoxia-inducible factor 1-alpha; DMT1, divalent metal transporter 1; ASL4, acyl-CoA synthetase long-chain family member 4; IL-6, interleukin 6; glutathione peroxidase 4; LOX, lipoxygenase; ROS, reactive oxygen species; FSP1, ferroptosis suppressor protein 1; Nrf2, nuclear factor erythroid 2-related factor 2 (Nrf2).

The association between ferroptosis and cerebral ischemia is complex. Several previous studies elucidated that the activation of ferroptosis following an ischemic episode involves iron overload (Tuo et al., 2017) and excitatory toxicity induced by the upregulation of xCT (one heterodimer composed of system xc-) (Cheah et al., 2006), as well as lipid peroxidation mediated by the overexpression of ACSL4 (Gubern et al., 2013) and 12/15-LOX (Yigitkanli et al., 2017; Figure 1).

### Mechanisms of Iron Overload During Cerebral Ischemia

Iron overload following IS is profoundly significant upon the activation of ferroptosis. Reportedly, iron overload after cerebral ischemia aggravated cell death in brain tissues, whereas blockade of iron overload elicited the opposite effect, protecting cerebral tissues from ischemic injury (Tuo et al., 2017).

Additionally, a study reported that serum levels of hepcidin and iron increased in IS patients. This indicates that hepcidin plays an important role in iron overload during cerebral ischemia (Petrova et al., 2016). The internalization and degradation of hepcidin are induced through its binding with FPN1 on the cell membrane, rendering it a negative regulator of iron release (Petrova et al., 2016). Currently, there are two potential pathways responsible for iron overload in ischemic brain tissue. The primary pathway involves increased expression of interleukin-6 (IL-6) owing to ischemia. IL-6 potentiates the expression of hepcidin through the JAK/STAT3 pathway, leading to a decrease in FPN1 and, consequently, reduced iron release. This results in intracellular iron overload (Ding et al., 2011). However, the mechanism of IL-6 upregulation in cerebral ischemia remains unclear. Similarly, the upregulation of hypoxia-inducible factor 1-alpha (HIF-1α) after cerebral ischemia results in iron overload, increasing transferrin receptor 1 (TFR1) expression. Considering that the TF-TFR1 pathway is potentially necessary for neurons to absorb iron, the increased iron uptake is possibly a decisive step for iron overload (Ke and Qian, 2007; Tang et al., 2008).

Moreover, inhibition of iron overload can reduce the damage caused by IS. In transient IS models, an intramuscular injection of the iron-chelating agent deferoxamine markedly reduces the volume of cerebral infarction (Xing et al., 2009). Another iron-chelating agent, 2,2-bipyridine, exerted a protective effect in a rat model of permanent cerebral ischemia (Demougeot et al., 2004). A specific iron-chelating agent, salicylaldehyde isonicotinoyl hydrazine (SIH), effectively alleviated ischemia–reperfusion injury (Tuo et al., 2017). Therefore, iron overload occurs in IS and is closely related to ferroptosis, implying that iron overload is an important regulatory mechanism of ferroptosis in IS.

### Iron-Related Proteins and IS

Recent studies revealed that TFR1 expression is upregulated in the brain during cerebral ischemia in rats, and Naotaifang extract reduces TFR1 expression to inhibit ferroptosis and improve brain injury (Lan et al., 2020). Paradoxically, some studies demonstrated that TFR has the opposite effect. Pretreatment with low-dose thrombin can increase the HIF-1α level through the p44/42 MAPK pathway, upregulating TF and TFR expression in the brain parenchyma and reducing iron-overload-mediated cerebral ischemic injury (Hua et al., 2003). This is because the brain TF can absorb iron from the circulation, and, additionally, iron can also quickly flow from the brain into the bloodstream (Zhang and Pardridge, 2001). This may suggest that the upregulation of TFR1 leads to iron overload, and accumulation of TFR1 beyond a certain threshold will allow iron clearance and reduce iron overload (Hua et al., 2003). Nevertheless, further investigations are imperative to elucidate the comprehensive effects of TFR1. Moreover, DMT1 expression was upregulated in rats with cerebral ischemia, and DMT1 inhibitors protected against ferroptosis, reducing cerebral ischemia injury (Lan et al., 2020).

## Amino Acid Metabolism and Cerebral Ischemia

### Expression of System xc- Is Upregulated in Cerebral Ischemia, Aggravating Injury

Notably, glutamate excitotoxicity after cerebral ischemia has been described as ferroptosis and can be effectively inhibited by the ferroptotic inhibitor ferrostatin-1 (Dixon et al., 2012). Moreover, glutamate excitotoxicity has been proposed as a mechanism of ferroptosis (Krzyżanowska et al., 2014). Signaling through glutamate receptors located on the neuronal cytomembrane, especially the N-methyl-D-aspartate receptor (NMDAR), promotes intracellular signaling and directly induces cell death (Cheah et al., 2006). NMDAR promotes calcium influx, extracellular iron uptake (including free iron and holotransferrin), and neuronal excitotoxicity through the NMDA-NO-Dexras1-PAP7-DMT1 pathway (Cheah et al., 2006).

System xc- is a heterodimer composed of SLC3A2 (regulatory subunit) and SLC7A11 (also known as xCT), playing a key role in the amino acid transport system (Dixon et al., 2012). In most animal models of neurological diseases, xCT is upregulated and leads to the release of non-synaptic glutamate, which in turn activates glutamate receptors. Excitotoxicity induced by xCT through glutamate receptors circumvents the antioxidant protection afforded by system xc- itself (Lewerenz et al., 2013). After the *in vitro* oxygen-glucose deprivation of neurons, HIF-1α binds to the promoter of xCT, upregulating its expression and then inducing injury (Hsieh et al., 2017). Conversely, the downregulation of xCT expression by N-acetylcysteine and ceftriaxone significantly reduced IS injury (Krzyżanowska et al., 2017). Neuronal excitotoxicity, also called ferroptosis, damages ischemic tissue through NMDAR activation, mediated by an increased extracellular glutamate content, mainly caused xCT after cerebral ischemia; repression of system xc- can inhibit ferroptosis to prevent damage. However, deletion of the transport subunit of system xc- does not impair the development of a young mouse brain and exerts only a marginal impact on an adult mouse brain (Sato et al., 2005), possibly induced by the presence of a substance compensating for system xc- *in vivo*. This rationalizes that an increased expression of system xc- in cerebral ischemia cannot inhibit but can promote the occurrence of ferroptosis. Intriguingly, a study has demonstrated that increased expression of the *p53* gene in rats repressed xCT and resulted in ferroptosis during cerebral ischemia (Lan et al., 2020). These seemingly contradictory results suggest that there exists an intricate relationship between xCT, cerebral ischemia, and ferroptosis. Therefore, additional research in this direction is needed in the future.

### Upregulation of GPX4 and GSH Can Improve Cerebral Ischemic Injury

GPX4 and GSH are endogenous inhibitors of ferroptosis, and their content is closely related to cerebral ischemia. Presently, it is believed that the loss of GPX4 function is crucial for ferroptosis owing to its pivotal role in the inhibition of lipid peroxidation (Stockwell et al., 2017). Furthermore, the inhibition of ferroptosis by GPX4 suppression reduced brain damage in rats subjected to ischemic damage. In ischemic rat models, significantly low levels of GPX4 resulted in ferroptosis, whereas high levels of GPX4 could improve brain injury (Lan et al., 2020). Selenium promotes the expression of GPX4 by activating the transcription factors TFAP2c and Sp1, effectively inhibiting ferroptosis, and alleviating IS injury (Alim et al., 2019). Carvacrol, a drug indicated for the treatment of cerebral ischemia, is beneficial in inhibiting ferroptosis in gerbils by potentiating the expression of GPX4 and attenuating hippocampal neuronal damage during cerebral ischemia–reperfusion injury (Guan et al., 2019).

GSH is essential for GPX4 activity; furthermore, it is the only intracellular ligand of Fe^2+^ that prevents the generation of highly toxic hydroxyl radicals, thereby inhibiting ferroptosis (Philpott and Ryu, 2014). Moreover, promoting GSH generation is beneficial for ameliorating IS. As a clinically approved drug for acute IS, edaravone has been proposed to combat ferroptosis, especially in cystine deficiency (Homma et al., 2019). In summary, enhanced GPX4 expression and GSH synthesis can suppress ferroptosis and mitigate ischemic brain damage. However, currently, researchers are focusing on the effect of GPX4 and GSH rather than on the upstream mechanism of cerebral ischemia.

## Lipid Metabolism and Cerebral Ischemia

### Inhibition of ACSL4 Inhibits Ferroptosis and Improves Cerebral Ischemic Injury

ACSL4 and LPCAT3 are involved in the remodeling of phosphatidylethanolamine, which affects the synthesis of lipid peroxides. However, only ACSL4 is upregulated and participates in ischemic injury. Reportedly, the inhibition of ACSL4 can prevent ferroptosis; thus, cell death and intestinal injury are alleviated (Li et al., 2019). Additionally, ACSL4 is widely expressed in brain tissues and is increased during cerebral ischemia, with an increased expression of miRNA-347 upregulating ACSL4 at the post-transcriptional level, and mediating neuronal death (Gubern et al., 2013). Rosiglitazone can selectively repress the activity of ACSL4, thereby preventing ferroptosis in the neurons of cerebral ischemic models and protecting brain function (Sayan-Ozacmak et al., 2012). In summary, the upregulation of ACSL4 after cerebral ischemia induces ferroptosis in neurons, leading to IS injury.

### Inhibition of Lox Can Inhibit Ferroptosis and Improve Cerebral Ischemic Injury

LOX is the key enzyme catalyzing PUFAs to initiate lipid peroxidation and ferroptosis (Lei et al., 2019), highly expressed after cerebral ischemia, and LOX inhibitors can suppress ferroptosis (Lei et al., 2019) to attenuate injury (Karatas et al., 2018). One LOX inhibitor, called ML351, exerted a protective effect in cerebral ischemia–reperfusion injury (Tuo et al., 2017). Furthermore, there are several subtypes of LOX, including 12/15-LOX. In animal models of brain ischemia, 12/15-LOX expression increases, and the inhibition of 12/15-LOX can reduce the rate of the neuronal death, improving recovery (Karatas et al., 2018). Previous studies have shown that suppressing 12/15-LOX can result in the inhibition of ferroptosis (Li et al., 2018). To date, it remains unclear whether multiple subtypes work in conjunction or whether only one subtype is sufficient to initiate lipid peroxidation and ferroptosis, necessitating further study.

In conclusion, the mechanism of ferroptosis during cerebral ischemia is related to the accumulation of intracellular Fe^2+^, lipid peroxides, and oxidative damage induced by the downregulation of GPX4. The specific factors are as follows: the increased expression of HIF-1α enhances the expression of TFR1 and xCT, both of which potentiate iron uptake and iron overload; increased IL-6 induces an increase in hepcidin content, which suppresses iron excretion and leads to iron overload; an unknown mechanism triggers the downregulation of GPX4; and increased expression of ACSL4 and 12/15-LOX leads to lipid peroxidation in the cell membrane.

## Other Mechanisms: Inhibition of Ferroptosis by Nrf2 Is Expected to Improve Is Injury

Nuclear factor erythroid-2-related factor 2 (Nrf2) is an important regulator of the cellular antioxidant defense system. A study demonstrated that the appropriate activation of Nrf2 promotes the alleviation of cerebral ischemic injury (Ma, 2013). Several genes targeted by Nrf2 are strongly associated with the progression of ferroptosis, including ferritin heavy chain 1, FPN1, GSH, and GPX4 (Ma, 2013; Kerins and Ooi, 2018). Furthermore, it has been clarified that the expression level of Nrf2 is dependent on ferroptosis sensitivity. The enhanced expression of Nrf2 can inhibit ferroptosis. Conversely, the inhibition of Nrf2 expression induces ferroptosis (Sun et al., 2016). Nrf2 inhibits ferroptosis by promoting the expression of GSH and GPX4, thus enhancing antioxidant function. Moreover, Nrf2 promotes the expression of ferritin and FPN1 to store and secrete free iron concurrently, thus reducing the accumulation of iron in cells and preventing ferroptosis (Yang et al., 2017). Therefore, Nrf2 is a promising anti-ferroptosis target.

Currently, evidence clarifying the mechanism by which Nrf2 directly affects ferroptosis during stroke is scarce. However, there are several indications regarding the role of the Nrf2 pathway in IS. Reportedly, epicatechin can cross the blood–brain barrier to exert a protective effect in transient IS through the Nrf2 pathway (Shah et al., 2010). However, related laboratory and clinical data elucidating this mechanism remain lacking.

## Concluding Remarks and Perspectives

Cerebral ischemia can induce ferroptosis and, in turn, ferroptosis aggravates cerebral ischemic injury; inhibiting ferroptosis can alleviate this injury. However, the regulatory mechanisms involved with ferroptosis in cerebral ischemia, including iron overload and regulatory mechanisms upstream of GPX4, 12/15-LOX, and Nrf2, remain unclear; in particular, whether ferroptosis causes cell death directly or by other means requires further investigation. Additionally, the relationship between ferroptosis and other cell death mechanisms, such as apoptosis and pyroptosis, in cerebral ischemia, is an important direction for future research. The resolution of these obstacles will provide crucial support for the development of ferroptosis as a target in cerebral ischemia interventions and an important direction for the study of pathological mechanisms involved in cerebral ischemia.

## Author Contributions

XS and BT reviewed the literature and drafted the manuscript. BL, HT, and BT finalized the manuscript and provided suggestions for improvements. All authors participated in designing the concept of this manuscript.

## Conflict of Interest

The authors declare that the research was conducted in the absence of any commercial or financial relationships that could be construed as a potential conflict of interest.

## Abbreviations

12/15-LOX: 12/15-lipoxygenase
AA: arachidonic acid
ACSL4: acyl-CoA synthetase long-chain family member 4
AdA: adrenic acid
CEF: ceftriaxone-
CoQ_10_: coenzyme Q_10_
CoQ_10_H_2_: reduced coenzyme Q_10_
DAMP: damage-associated molecular pattern
DMT1: divalent metal transporter 1
FPN: ferroportin1
FSP1: ferroptosis suppressor protein 1
FT: ferritin
GPX4: glutathione peroxidase 4
GSH: reduced glutathione
GSSG: oxidized glutathione
HIF-1 α: hypoxia-inducible factor-1 α
HTF: holotransferrin
IS: ischemic stroke
LIP: labile iron pool
L-O: alkyl oxygen free radicals
L-OH: phospholipids-H
L-OOH: phospholipids-OH
LOX: lipoxygenase
LPCAT3: lysophosphatidylcholine acyltransferase 3
miR: miRNA
MVA: mevalonate
NAC: N-acetylcysteine
NADPH: nicotinamide adenine dinucleotide phosphate
NMDAR: N-methyl -D-aspartate receptor
Nrf2: nuclear factor erythroid-2-related factor 2
PE: phosphatidylethanolamine
PUFAs: polyunsaturated fatty acids
ROS: reactive oxygen species
system xc-: cystine/glutamate reverse transporters
SIH: salicylaldehyde isonicotinoyl hydrazine
STEAP3: six-transmembrane epithelial antigen of the prostate 3
TF: transferrin
TFR1: transferrin receptor 1.
